# Unique Hyperspectral Response Design in High-Speed Photodetectors Enabled by Periodic Surface Textures

**DOI:** 10.21203/rs.3.rs-3140578/v1

**Published:** 2023-07-21

**Authors:** Ahasan Ahamed, Amita Rawat, Ahmed S. Mayet, Lisa N McPhillips, M Saif Islam

**Affiliations:** Electrical and Computer Engineering, University of California Davis, 1207 Kemper, Davis, 95616, CA, USA

**Keywords:** Avalanche photodiodes, hyperspectral imaging, multispectral imaging, photon-trapping features, spectral response engineering

## Abstract

Engineered spectral response in photodetectors combined with advanced signal processing and deep learning-based image reconstruction enables widespread applications of hyperspectral imaging. These advancements in spectral imaging eliminate the need for complex filters and dispersion lenses, benefiting various fields such as remote sensing, astronomy, agriculture, healthcare, forensics, food quality assessment, environmental monitoring, and cultural heritage preservation. We present a spectral response design method using photon-trapping surface textures (PTSTs) to enable system miniaturization by eliminating the need for external diffraction optics and employing detector-only spectral sensors. We additionally demonstrate the fabrication of cost-effective, high-performance silicon photodetectors with unique spectral responses by integrating PTSTs. These CMOS-compatible photodetectors are ultra-fast, highly sensitive, and suitable for wideband multi/hyperspectral imaging systems. Our investigation uncovers a prominent linear correlation between the PTST periods and the peak coupling wavelengths while observing a weaker relationship with the PTST diameters. Furthermore, we establish a significant association between inter-PTST spacing and wave propagation patterns. In a proof-of-principle demonstration, we effectively employ these photodetectors with distinct spectral responses to capture visible and near-infrared wavelengths for multispectral imaging. These findings support the feasibility of integrating high-performance on-chip spectrometers, offering compact form factors, extensive applicability, and real-time data acquisition and manipulation capabilities.

## 1 Main

Applications of spectrometers such as forensic science, art conservation, pharmaceutical imaging, molecular imaging, environmental contamination monitoring [1–8], etc., have been a major driving force toward the development of the miniaturized spectrometer and spectral response engineering. The very first use of prismatic and diffraction spectra in telescopic imaging was demonstrated by Fraunhofer, et al. back in 1898 CE [9]. Hyper/Multispectral imaging (HSI/MSI) with large form factor spectrometers have been extensively used for astrophysics and military applications for the past few decades [10, 11]. Lately, the HSI and MSI technologies are being exploited for biological tissue, molecules, and functional nanomaterial [12] analysis. Proteins, such as hemoglobin, melanin, etc., present in healthy versus infected tissues, interact uniquely when exposed to the optical stimulus that requires a wavelength-resolved, efficient, and fast capture of reflected, fluorescent, and transmitted electromagnetic (EM) waves facilitated by HSI/MSI imaging technology. Fourier transform infrared Spectroscopy exhibits exceptional wavelength resolution and a rapid response time [13]. However, a bulky measurement setup and excessive power consumption limit the scaling, portability, and approachability of the system. Advancements in fabrication technology have enabled miniaturized multi-spectral imaging and spectral response engineering [14]. Approaches such as the use of broadband diffractive and charge-coupled-devices in combination with a novel spectrum extraction algorithm [15], evanescently coupled spiral waveguides on silicon-on-insulator (SOI) substrates coupled with detector arrays [16], etalon (i.e., two semi-reflecting surfaces separated by an optically transparent medium) array [17], photonic molecules composed of micro-ring resonators [18], metasurface based imaging [19, 20], inverse design for broadband engineering [21], and surface gratings [22] are a few successful approaches demonstrating miniaturized spectrometers and computational imaging systems. However, challenges such as large device footprints, inefficient power consumption, complex peripheral circuitry, and the need for wavelength splitters and filters hinder the further scaling of these systems.

**Fig. 1:**
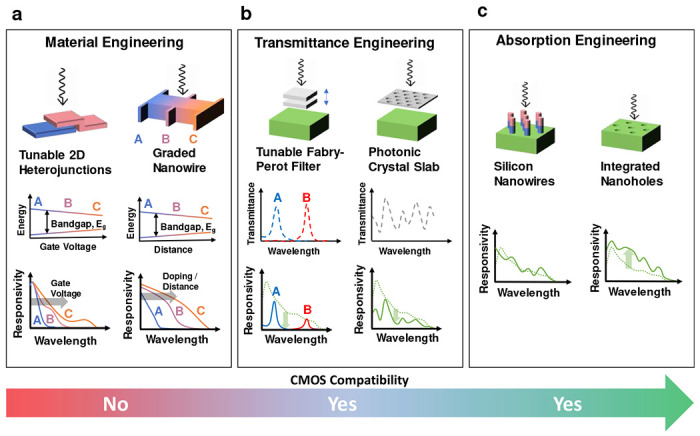
Various approaches used thus far for spectral response engineering: An illustration of spectral response engineering method (top) and their respective responsivity over wavelength (bottom). **a,** A gradual bandgap modulation is enabled by gate voltage-controlled tunable two-dimensional (2D) material heterojunctions [36] (left) and gradual doping [27] or quantum scale width manipulation in semiconductor [29] material (right) allowing distinct responsivity profiles. These methods involve CMOS-incompatible exotic 2D material processing or complex growth processes. **b,** Engineering transmittance of the devices by voltage-controlled thickness modulation of the Fabry–Pérot resonator filter [37] (left) and photonic crystal slabs [38] (right). This method is compatible with the CMOS technology, however, results in reduced responsivity of the detectors. **c,** Engineering absorption by using nanowire-based photodetectors [31] (left) and integrated nanoholes in photodetectors [34] (right). The introduction of nanostructures such as pillars or surface textures such as nano/micro holes is CMOS compatible and uniquely enhances the light-matter interaction for each wavelength translating into unique responsivity.

Recent advancements in spectrometer-on-chip technologies have explored engineered spectral responses combined with artificial intelligence (AI)-assisted image reconstruction techniques [23–25]. Approaches such as plasmonic surface structure-based spectral response modulation [26], band gap engineering by alloying [27] or supper-lattice stacking [28, 29], transmission engineering [30], nanowire array-based absorption engineering [31], metasurfaces [20], etc., have shown promising results. A compilation of available spectral response engineering approaches is presented in Fig. 1. Despite remarkable performances, these methods remain challenging to be integrated into the complementary metal oxide semiconductor (CMOS) process line due to complex fabrication processes, usage of exotic materials, and non-scalable device design. The incorporation of surface texturing (ST) to enhance the EM wave absorption and to manipulate the light-matter interaction have been explored in great detail [32–35], however, their potential in HSI/MSI applications remains unexplored.

This study demonstrates the utilization of photon-trapping surface textures (PTST) in photodetectors for absorption spectral response engineering. A detailed method to design the PTST is presented, enabling unique spectral responses by tuning the PT hole diameter (*d*) and periodicity (*p*). We have established a mathematical model predicting the spectral response in terms of the electromagnetic wave coupling under the influence of PTST. Further, we fabricate a variety of PTST-equipped photodetectors to show unique absorption spectra in the form of unique external quantum efficiency (EQE). We showcase an astute match between the results predicted from the finite-difference time-domain (FDTD) simulations and the analytical model, and the results generated from the experiments. Finally, we present proof-of-principle hyperspectral imaging using the absorption spectra of a unit PTST-equipped photodetector. The proposed device design method allows a controlled spectral response engineering essential for AI-assisted computational imaging and the analytical model can further be used to build compact models of PTST-equipped photodetectors for large-scale circuit designing.

## 2 Results

**Fig. 2:**
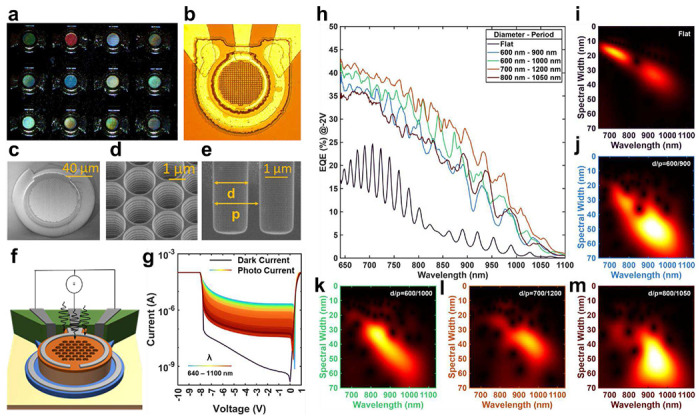
Fabricated photodetectors with unique spectral response: **a,b,** An optical micrograph of the fabricated photodetectors with PTST features showcasing iridescence under white light evincing their unique responsivity. **c-e,** The SEM images of the device and the introduced PTST features. The use of ICPRIE has resulted in smooth and straight PTST sidewalls essential to minimize surface scattering. **f,** A schematic depiction of the device fabricated and applied electrical and optical stimuli. **g,** The DC I-V characteristics of the photodetector are in the dark and under the illumination of laser wavelengths ranging from 640-1100 nm. **h,** The unique EQEs of four photodetectors with PTST are compared against that of a flat device for a range of illumination wavelength at a fixed laser power of 10 μW and an applied bias of −2V. The legend depicts the PTST hole diameter and period. The EQE of the flat device shows resonance due to the Fabry-Pérot cavity effect, while the devices with PTST are showing enhanced EQE with unique spectral responses. **i-m,** Corresponding Morlet wavelet transforms (color-coded axes) for each photodetector showcasing their unique responsivity. The x-axis plots illumination wavelengths while the y-axis depicts the spectral width of the respective crest and trough. The subtle uniqueness in the 2D EQE spectral response is prominently evident in the Morlet wavelet transforms.

We have engineered the absorption spectral response by incorporating photon-trapping surface textures (PTST) into the photodetectors. The devices are fabricated by using CMOS-compatible processes on a silicon-on-insulator wafer. The die consists of a variety of PTST dimensions where the PT hole diameter range varies from 600 to 1500 nm and the period range varies from 900 to 3000 nm. The details for the device fabrication process steps are presented in the supplementary document. The devices exhibit iridescent colors due to varying PTST dimensions, as shown in the microscopic images of Fig. 2a,b. In Fig. 2c–e, we show scanning electron microscopic (SEM) images of the PTST. The line-of-sight etching process enabled by inductively coupled reactive ion etching (ICPRIE) results in straight side walls that help reduce the surface scattering of the EM waves. Fig. 2f showcases the device characterization bias arrangement. A semiconductor parameter analyzer is used to apply a Direct-Current (DC) bias voltage ranging from 0-1 V in the forward bias, and 0-10 V in the reverse bias. NKT supercontinuum laser source and a wavelength filter are used to vary the illumination wavelength. The captured DC current-voltage (I-V) profile for a range of illumination wavelengths (640-1100 nm) at a fixed illumination power of 10 μW is shown in Fig. 2g. The I-V trends under illumination are compared against the dark I-V profile. The devices’ transient response and multiplication gain performance are presented in the supplementary document. Fig. 2h, presents the external quantum efficiency (EQE) trends measured from four different PTST-equipped photodetectors and compared against that of a flat (without-PTST) photodetector. The EQE of a flat device possesses regular fringes as an outcome of Fabry–Pérot cavity resonance due to the presence of SOI substrate [34]. The introduction of PTST substantially diminishes this Fabry-Pérot cavity (discussed in the Supplementary document), and significantly enhances EQE owing to the photon-trapping technique [32, 34, 39]. Additionally, each device with PTST exhibits a distinct EQE response attributed to unique EM wave interaction with various PTST feature sizes. In Fig. 2i–m, we showcase the Morlet wavelet transform of the EQE profile to highlight the influence of PTST on illumination wavelength absorption and corresponding spectral width resulting in their respective EQE trend. The apparent uniqueness in the EQE trends of various PTST-equipped photodetectors shown in Fig. 2h have been made prominent in their respective wavelet transform contour plots.

**Fig. 3:**
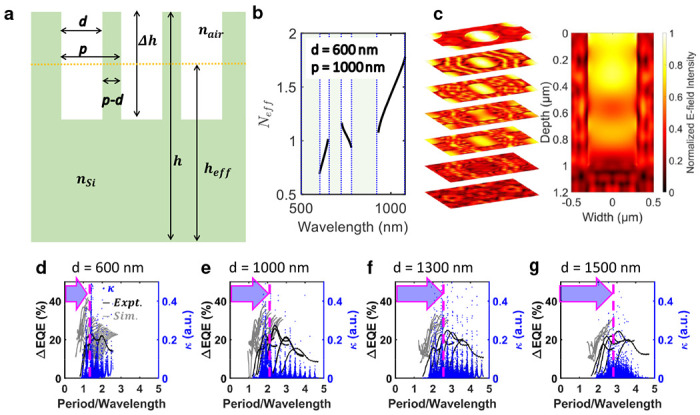
Mathematical formulation of the light-matter-interaction: **a,** Simplified 2D cross-section of PTST used for the analytical model; **b,** The effective refractive index ($N_{eff}$) for *d* = 600 nm and *p* = 1000 nm PTST arrangement. The voids in the $N_{eff}$ represent photonic band gap formation; **c,** TE mode propagation profile shown for a unit-cell of the PTST array. **d-g,** A comparison of experimentally measured external absorption efficiency (i.e., EQE in black), simulated internal absorption efficiency (i.e., IQE in gray) on FDTD Lumerical platform, and analytically calculated coupling coefficient (*κ* in blue) as a function of PTST period and illumination wavelengths for **d,** 600 nm, **e,** 1000 nm, **f,** 1200 nm, and **g,** 1500 nm PTST diameters. The Period/Wavelength with respect to the peak absorption efficiency and coupling increases as the diameter of the PTST increases from 600 nm to 1500 nm. For smaller PTST diameters, the PTST period comparable to the illumination wavelength result in maximum coupling and absorption efficiency; whereas, for larger PTST diameters the shorter EM wavelength show better coupling.

In Fig. 3a, we present a simplified 2D cross-section of the PTST array introduced into Si to build an analytical model for EM wave coupling. Fig. 3b shows the effective refractive index ($N_{eff}$) calculated for a fixed PTST dimension (diameter, *d*: 600 nm; period, *p*: 900 nm) as a function of ωp/c (ω=2πf, where *f* is EM wave frequency; *c* is the speed of light). The discontinuity (presence of voids) in the $N_{eff}$ trend represents the photonic bandgap formation [40] with the introduction of PTST that selectively forbids the coupling for certain wavelengths and results in a weak coupling (a weak intrinsic absorption) governed by the electronic bandgap of the material. A real $N_{eff}$ results in a strong EM wave coupling. The EQE trend modulation with introducing the PTST array into the photodetectors is an outcome of unique EM wave interaction with different PTST dimensions (i.e., diameter, *d*, and periodicity *p*). The *d* and the *p* dimensions are of the order of the illumination wavelengths which result in photonic bandgap formation and a non-uniform absorption of the EM wave. Figure 3c shows the trans electric (TE) mode propagation in a PTST-equipped device simulated in the Lumerical FDTD platform. In Fig. 3d–g, we analytically calculate the EM wave coupling coefficient (*κ*) for a range of PTST dimensions and illumination wavelengths and present a good match against the experimental and simulated ΔEQE (a proportionate measure of the increased absorption due to the coupling) trend. We show that a wider PTST diameter requires a larger PTST period per unit wavelength for better coupling, i.e., for a fixed PTST period, a denser PTST array will enable stronger coupling for shorter wavelengths. Such controlled interaction of EM waves enabled by PTST facilitates a framework for spectral response engineering.

### 2.1 Coupling analysis

**Fig. 4:**
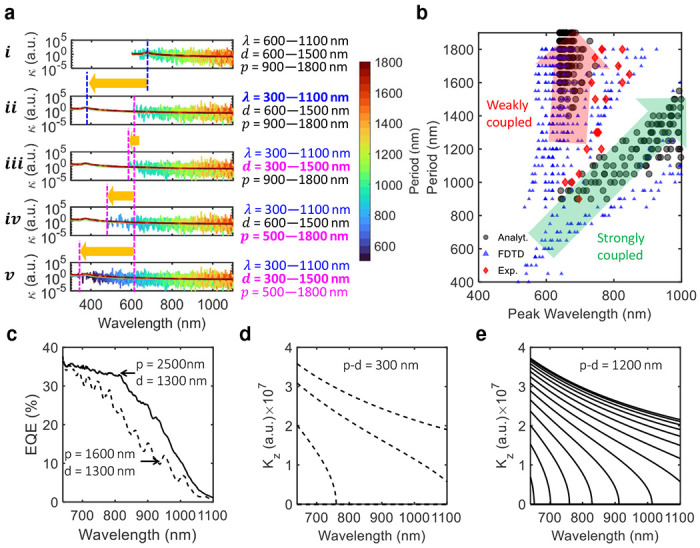
Coupling and fringes: **a,** Coupling coefficient versus wavelength trends compared against five different combinations of *λ*, *d*, and *p* range from **(*i*)** to **(*v*)**. In **(*i*)** *κ* is plotted against wavelength for a range of *λ*, *d*, and *p* aligned with that of experiments. The presence of a solid monotonous trend of weakly coupled scenarios overlapped with strongly coupled spikes showcasing the dual coupling nature. Changing the range of *λ* to 300–1100 nm in **(*ii*)** only enables the lower wavelengths to exhibit a weak intrinsic absorption-driven coupling, whereas, the strong coupling remains intact as the *d* and *p* range. Further changing the range of *d* in **(*iii*)** marginally enables a strong coupling at lower wavelengths, and changing the range of *p* to 500–1800 nm results in a significant increase in the strong coupling at lower wavelengths in **(*iv*)**. Finally, changing the range of both *d* and *p* to 300–1500 nm and 500–1800 nm respectively allows a strong coupling to occur even at further lower wavelengths in **(*v*)**. **b,** A comparison of Peak coupling wavelength versus PTST period among experimental, FDTD simulation, and analytical trends. Analytical formulation suggests a linear relationship of the peak wavelength with the PTST period which is also reflected in simulation and experimental data (the green guiding arrow). The analytically predicted weakly coupled scenarios are evident in the experimental and FDTD simulation results (the red guiding arrow). **c-e,** Allowable lateral TE modes propagation in the *p* − *d* widths of 300 nm and 1200 nm respectively. EQE spectrum versus wavelength plot **c,** for the aforementioned *p*−*d* differences in the PTST-equipped photodetectors. A low number of allowable TE modes in the dispersion plot for *p* − *d* = 300 nm **d,**, results in higher fringes as evident in the EQE profile. An increased number of allowable TE modes in the dispersion plot for *p* − *d* = 1200 nm **e,**, corresponds to reduced fringes in the EQE spectrum due to the averaging effect.

Due to the PTST-governed photonic bandgap formation, there exist weakly coupled and strongly coupled light-matter interaction scenarios [41]. The weakly coupled scenario follows the intrinsic absorption characteristics of the material (i.e., Silicon), whereas, the strongly coupled scenarios exhibit PTST-dependent EM wave coupling in addition to the intrinsic absorption. In Fig. 4a, we have plotted the coupling coefficient (*κ*) calculated from Eq. (2) by changing the range of *λ*, *d*, and *p*. We show that the *κ* exhibits the aforementioned coupling scenarios, a weak intrinsic absorption-driven coupling plotted in a solid monotonous trend, and strongly coupled scenarios plotted as overlapping spikes in Fig. 4a. The series of figures from (*i*) to (*v*) present the weak and the strong coupling behavior by manipulating the range of *λ*, *d*, and *p*. Fig. 4a,i represents the baseline *κ* profile in alignment with the experiments, i.e., the range of *λ*, *d*, *p* are 600–1100 nm, 600–1500 nm, and 900–1800 nm respectively, are in alignment with the experiments. In Fig. 4a,ii, we have reduced the lower bound of *λ* to 300 nm while keeping *d* and *p* range fixed. Doing so only impacts the weakly coupled scenarios while keeping the strongly coupled scenarios intact. This shows that the strongly coupled scenarios are strictly governed by PTST dimensions, i.e., *d* and *p*. In Fig. 4a,iii, we have reduced the lower bound of *d* to 300 nm while keeping the range of *λ* and *p* consistent with Fig. 4a,ii. The introduction of lower *d* marginally allows lower wavelengths to undergo strong coupling. Further, we reduced the lower bound of *p* to 500 nm while keeping the range of *λ* and *d* consistent with Fig. 4a,ii. This further allows significantly lower wavelengths to undergo strong coupling as shown in Fig. 4a,iv. Finally, we reduced the lower bounds of both *d* and *p* to 300 nm and 500 nm, which results in a strong coupling throughout the wavelength range (Fig. 4a,v). This exercise concludes that the weakly coupled wavelengths are purely material-dependent and governed by the intrinsic absorption coefficient. The strongly coupled scenarios are governed by the interplay of *d* and *p* with a strong dependence on the *p*. To establish a qualitative relationship between the EM wave coupling and PTST dimensions, we have extracted the wavelengths at which the peak coupling occurs for a range of *d* and *p*. The extracted peak coupling wavelengths exhibit two distinct slopes representing a weak and a strong coupling as shown in Fig. 4b. The weakly coupled peak wavelengths are bounded by the lower bound of the wavelength range used in the analysis. The peak wavelengths in the strongly coupled scenario exhibit a fairly linear relationship with the period, *p*. Further detail of the analysis is presented in the supplementary document and supplementary video. Furthermore, we have validated the analytically generated period versus peak wavelength trend with FDTD simulations and experimental results. We observe the presence of weakly coupled and strongly coupled scenarios in the simulations and experiments as predicted by the analytical model as shown in Fig. 4b.

### 2.2 TE mode analysis

In Fig. 4c, we present two sets of EQE spectrum for two different PTST periods (1600 nm, and 2500 nm) for a fixed PTST diameter (1300 nm). We show that the device with *p* − *d* = 300 nm (i.e., *d* = 1300 nm; *p* = 1600 nm), has prominent fringes in its EQE spectrum. However, the device with *p* − *d* = 1200 nm (i.e., *d* = 1300 nm; *p* = 2500 nm) has a relatively smooth EQE spectrum. The former device (*p* − *d* = 300 nm) has only a few possible TE modes propagating in the *p* − *d* region as shown in Fig. 4d, as opposed to the latter device (*p* − *d* = 1200 nm) with an increased number of TE modes propagating in the *p* − *d* region as shown in Fig. 4e. Increased number of TE modes propagation results in a smooth EQE trend due to the averaging effect, where, as a few TE modes result in fringes in the EQE profile. The fringes in the EQE spectra can be manipulated by selecting a narrower or a wider *p* − *d* width in PTST-equipped photodiodes. A detailed explanation of the propagated TE modes is shown in the supplementary document.

### 2.3 Performance benchmarking

We present a thorough device performance benchmarking based on device form factor, wavelength range covered, and compatibility with existing CMOS processes in Table 1. Most of these exceptionally good spectrometer approaches are not compatible with the CMOS processes, and a few CMOS-compatible approaches require a larger form factor. The devices with engineered spectral responses proposed in this work show a wide wavelength range coverage and a smaller device footprint, and the device fabrication processes used are aligned with the CMOS foundry.

### 2.4 A proof-of-principle demonstration

**Fig. 5:**
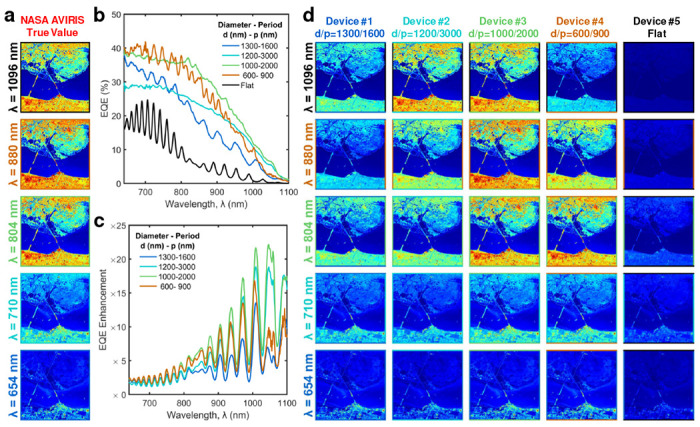
Proof-of-principle demonstration: **a,** HSI of Kennedy Space Center captured by NASA AVIRIS is shown at wavelength 654 nm, 710 nm, 804 nm, 880 nm, and 1096 nm respectively. **b,** EQE and **c,** ΔEQE comparison among silicon photodetectors with integrated PTST with varying diameters, *d* and periods, *p*, and a flat device. **d,** Simulated HSI image prediction of Kennedy Space Station for the same wavelengths (654 nm, 710 nm, 804 nm, 880 nm, and 1096 nm) using four unique PTST-equipped devices and a flat device. The PTST-enabled wavelength selective imaging allows efficient capture of finer details of the frame on a silicon platform.

To demonstrate the application of these unique response photodetectors we have used the Hyperspectral imaging (HSI) dataset captured for the Kennedy Space Center (KSC) by NASA AVIRIS, and unit device performance to predict the image sensor array performance. The image sensors used in AVIRIS are InSb-based photodetectors. We have chosen 654 nm, 710 nm, 804 nm, 880 nm, and 1096 nm wavelength images captured by AVIRIS, and false color images at each wavelength are shown in Fig. 5a. In this study, we predict the image formation at each wavelength using Si-based flat and PTST-equipped photodetectors. Comparison of the EQE and ΔEQE trends for flat (without PTST) and PTST-equipped devices are shown in Fig. 5b,c. The introduction of the PTST array into the photodetector increases the EQE over a range of wavelength spectra based on the PTST dimensions and results in a greater contrast as against the flat photodetector as shown in Fig. 5d enabling CMOS-compatible near-infrared HSI capabilities that can be further enhanced by AI-assisted image processing algorithms.

## 3 Conclusion

We present the design and fabrication of efficient, high-speed, high-gain photodetectors with unique spectral responses by introducing photon-trapping surface textures (PTST). We further present an analytical formulation of electromagnetic (EM) wavelength coupling as a function of PTST dimensions. We show that the peak coupling wavelength exhibits a linear relationship with the PTST period, *p*, and a weak dependence on the PTST diameter, *d*. We also show that the *p* − *d* value controls the extent of fringes in the absorption efficiency of the device. These PTST-equipped photodetectors and their analytically explained EM-wave interaction can potentially transform unique spectral response engineering. The unique spectral responses through PTST incorporation will enable AI-assisted image reconstruction, along with an opportunity for extreme miniaturization and on-chip integration of spectrometers facilitating a pivotal step forward in the realization of the on-chip high-performance hyperspectral imaging systems on the silicon platform.

## 4 Methodology

### 4.1 Formulation of the coupling coefficient

An intuitive physics-based light-matter-interaction understanding is essential for accurate spectral response engineering. In this section, we present an analytical formulation of the EM wave interaction with the introduced PTST holes in the form of coupling coefficient, *κ*[40, 47, 48]. The EM wave coupling nature with various PTST dimensions is validated against the experiments and simulations.


(1) $$N_{eff}=\frac{c}{\omega p}\cos^{-1}\{\cos\left(\frac{n_{air}\omega d}{c}\right)\cos\left(\frac{n_{Si}\omega \left(p-d\right)}{c}\right)\;\;\;-\frac{n_{air}^{2}+n_{Si}^{2}}{2n_{air}n_{Si}}\text{sin}\left(\frac{n_{air}\omega d}{c}\right)\sin\left(\frac{n_{Si}\omega \left(p-d\right)}{c}\right)\}$$

Equation (1) presents an analytical formulation of the effective refractive index ($N_{eff}$) of Si after introducing the PTST [40]; where, ω=2πf is the angular frequency, *c* is the speed of light in free space, *d* and *p* are PTST diameter and periodicity respectively, and $n_{air}$ and $n_{Si}$ are refractive indexes of air and Si respectively.

(2) $$\kappa =\frac{\pi }{\lambda }\times \frac{\text{Δ}h}{h_{eff}}\times \frac{n_{Si}^{2}-N_{eff}^{2}}{N_{eff}}\times \rho_{c}$$

where,

$$\rho_{c}=\frac{1}{2}\times \left(\frac{n_{Si}^{2}}{n_{air}^{2}}+\frac{n_{air}^{2}}{n_{Si}^{2}}\right)\times \frac{\left(\frac{N_{eff}^{2}}{n_{Si}^{2}}+\frac{N_{eff}^{2}}{n_{air}^{2}}+1\right)}{\left(\frac{N_{eff}^{2}}{n_{Si}^{2}}+\frac{N_{eff}^{2}}{n_{air}^{2}}-1\right)}\;\text{and}\;h_{eff}=h-\frac{d}{p}\text{Δ}h$$

Further, using Equation (1), the expression for coupling coefficient, *κ* is formulated in Equation (2); where, $\rho_{c}$ is the reduction factor, $h_{eff}$ is the effective height of silicon as shown in Fig. 3a.

### 4.2 TE Model dispersion relation


(3) $$K_{z}=\sqrt{\omega^{2}\mu \epsilon -\frac{m\pi }{p-d}}$$

In the presence of a PTST array in the photodetector, the trans-electric (TE) and trans-magnetic (TM) mode propagation is limited by the width of *p* − *d* region as shown in Fig. 3a. In this work, we have only studied the TE mode propagation and its impact on the EQE. The EM wave is introduced in the Si absorption layer and the propagation is limited by the *p* − *d* region. The TE mode dispersion relation is given by Equation (3), where, *μ* and *ϵ* are magnetic permeability and electric permittivity of silicon, and *m* is an integer (In this formulation, we have assumed that the electric field terminates at the $n_{air}/n_{Si}$ interface).

## Figures and Tables

**Table 1:** Benchmarking table comparing the device performance against existing literature.

Ref.	Materials used	Wavelength range (nm)	Form Factor (μm^2^)	CMOS compatibility
2014[15]	Polychromate diffractive optics with CMOS sensors	300-2500	-	Not compatible
2016[16]	Evanescently coupled multimode Si spiral waveguide	1520-1522	π250^2^	Compatible
2017[17]	Fabry–Pérot etalons	400-900	500×500	Not compatible
2019[38]	Photonic crystal slab on CMOS sensors	550-750	32×32	Compatible
2019[31]	Silicon nanowire array	400-800	150×150	Compatible
2020[42]	Perovskite quantum dot filters & CCD	250-110	7e4×7e4	Not compatible
2021[43]	Single tunable black phosphorus detectors	2000-9000	9×16	Not compatible
2021[44]	plasmonic nanoholes array on glass with CMOS sensors	480-750	100×100	-
2022[45]	MoS_2_/WSe_2_	405-845	22×8	Not compatible
2022[46]	PbS colloidal quantum dots	400-1300	15×15	Not compatible
2022[36]	ReS_2_/Au/WSe_2_ heterojunction	1150-1470	~20×20	Not compatible
2023[18]	Photonic molecule	1500-1600	60×60	Compatible
**This work**	**Photon trapping surface textures in Si**	**640-1100**	π**10^2^**	**Compatible**

## Data Availability

All data needed to evaluate the conclusions in the paper are present in the main text or the supplementary materials. Correspondence and requests for materials should be addressed to M.S.I. (sislam@ucdavis.edu).
